# The diversity and ecological roles of *Penicillium* in intertidal zones

**DOI:** 10.1038/s41598-019-49966-5

**Published:** 2019-09-19

**Authors:** Myung Soo Park, Seung-Yoon Oh, Jonathan J. Fong, Jos Houbraken, Young Woon Lim

**Affiliations:** 1 School of Biological Sciences and Institute of Microbiology, Seoul National University, Seoul, 08826 South Korea; 2 Science Unit, Lingnan University, Tuen Mun, Hong Kong; 3 Westerdijk Fungal Biodiversity Centre, Uppsalalaan 8, 3584 CT Utrecht, Netherlands

**Keywords:** Microbial ecology, Microbial ecology, Taxonomy, Taxonomy, Fungi

## Abstract

Members of the genus *Penicillium* are commonly isolated from various terrestrial and marine environments, and play an important ecological role as a decomposer. To gain insight into the ecological role of *Penicillium* in intertidal zones, we investigated the *Penicillium* diversity and community structure using a culture-dependent technique and a culture independent metagenomic approach using ITS (ITS-NGS) and partial β-tubulin (*BenA*-NGS) as targets. The obtained isolates were tested for halotolerance, enzyme activity, and polycyclic aromatic hydrocarbons (PAHs) degradation. A total of 96 *Penicillium* species were identified from the investigated intertidal zones. Although the *BenA*-NGS method was efficient for detecting *Penicillium*, some species were only detected using conventional isolation and/or the ITS-NGS method. The *Penicillium* community displayed a significant degree of variation relative to season (summer and winter) and seaside (western and southern coast). Many *Penicillium* species isolated in this study exhibited cellulase and protease activity, and/or degradation of PAHs. These findings support the important role of *Penicillium* in the intertidal zone for nutrient recycling and pollutant degradation.

## Introduction

The genus *Penicillium* is commonly isolated from various terrestrial environments such as indoor environments, soil, and food, and is known to play an important ecological role as a decomposer^1–3^. Recently, *Penicillium* species have been reported from various marine environments such as sand, seawater, and macroalgae^4–7^ and have proven to be valuable biological resources due to producing secondary metabolites and enzymes^5,8,9^. This suggests that *Penicillium* also plays an important role in the marine environment. Previous studies of marine environments focused on screening for applicable enzymes as well as novel bioactive compounds through the isolation of *Penicillium* species^5,6,8,9^. Studying the diversity and ecological roles is needed to enhance our understanding of *Penicillium* in marine environments.

The application of culture-independent techniques has provided new insights into a greater diversity and ecological role of fungi in marine habitats^10–12^. When comparing a variety of molecular methods such as ribosomal RNA (rRNA) clone libraries, denaturing gradient gel electrophoresis (DGGE), and terminal restriction fragment length polymorphism (T-RFLP) analysis to culture-dependent method, higher fungal diversities have been reported for the latter from deep sea sediment^13^, sea cucumber farming systems^14^, and sponges^15^. Recently, metagenomic analysis using next generation sequencing (NGS) has been used to further understand microbial characteristics in environments and has shown its usefulness in discovering fungal diversity in marine environments such as coral^10^ and mangrove sediments^16^. Hurdles to overcome are that taxonomic assignment of sequence data depends on the quality and comprehensiveness of available databases, as well as the resolution of the molecular marker^17^. Three loci (18S nuclear ribosomal small subunit rRNA gene [SSU], 28S nuclear ribosomal large subunit rRNA gene [LSU], and internal transcribed spacer [ITS] region) have been used in metagenomic studies due to high PCR amplification and sequencing success^18–20^. However, these loci have low resolution for species identification in the genera *Aspergillus*, *Penicillium*, and *Trichoderma*. Specific protein-coding genes sequence are recommended for species identifications in these genera^3,21–23^.

Intertidal zones play an important role in the marine nutrient cycling, decomposition, harboring biodiversity, and pollutant degradation^24^. Microbes are essential in the intertidal zone for mineralization of organic matter and degradation of pollutants^25,26^. Given their roles and activities in marine environments, fungal communities in the intertidal zone may be distinctive and have important ecological functions. Although reports of *Penicillium* in marine environment are increasing, there is no comprehensive study on species diversity and what roles they play.

In this study, we investigated *Penicillium* in intertidal zones (mudflat and sand) in South Korea. *Penicillium* is a good model genus for investigating fungal diversity and functional roles in the environment because of its frequent occurrence, high enzyme activity, and ability to grow on artificial media. Reliable species identification is possible because a standardized method has been outlined to identify *Penicillium* at the species level using morphology and the β-tubulin (*BenA*) gene^3^. To understand *Penicillium* diversity, community structure, and ecological roles, we used and compared a variety of methods: a culture-dependent approach with three different media, and a culture-independent metagenomic approach using ITS and *BenA* as targets. To understand the ecological roles of *Penicillium* in intertidal zones, we evaluated the halotolerance, enzyme activities (extracellular endoglucanase, β-glucosidase, and protease), and polycyclic aromatic hydrocarbons (PAHs) degradation for cultured species.

## Results

### *Penicillium* diversity from culture-dependent approach

**Figure 1:**
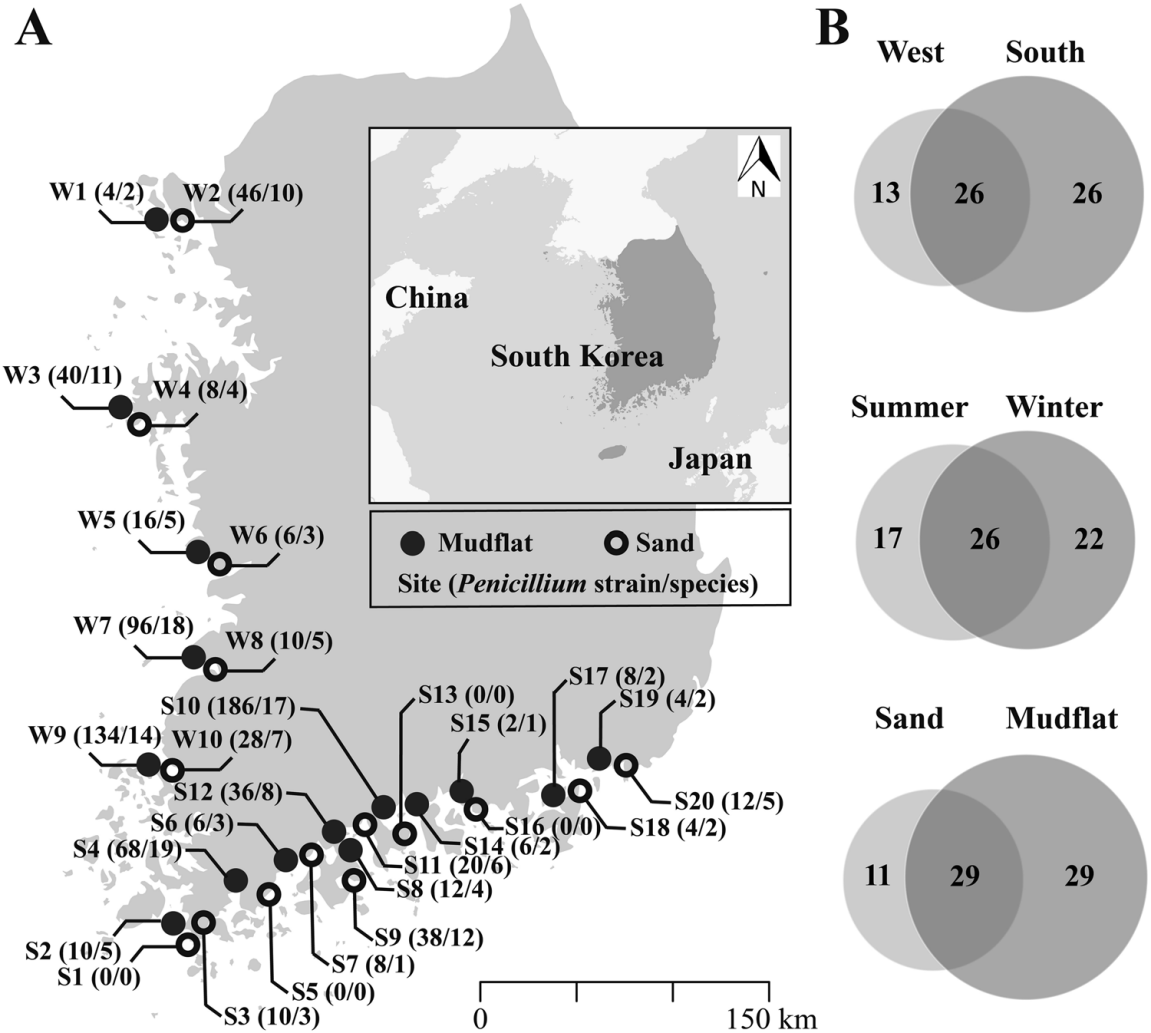
The number of identified *Penicillium* species from each sampling sites. (**A**) Map showing the number of (strains/species) at each location. (**B**) The number of *Penicillium* species isolated depended on season (summer and winter), seaside (western and southern coast), and substrate (mudflat and sand). Raw map was obtained from https://freevectormaps.com/ and edited in Adobe Illustrator CS6 (Adobe Systems Inc., CA, USA).

A total of 818 *Penicillium* strains were isolated from mudflat (626 strains) and sand (192 strains), collected along the western (388 strains) and southern coast (430 strains) of Korea (Table S1). On the basis of morphological comparisons and ITS sequences, 818 strains were categorized in 65 groups. One to three representative strains of each group were identified to the species level based on a section-by-section phylogenetic analysis using *BenA*, with a total of 187 strains chosen. These strains were identified as 57 species, with eight strains that could not be identified that represent eight potential new species. The species numbers of *Penicillium* differed depending on season, seaside, and substrate, although it was not statistically significant. The differences of *Penicillium* according to season, seaside, and substrate are shown in Fig. 1 and Table S2.

**Figure 2:**
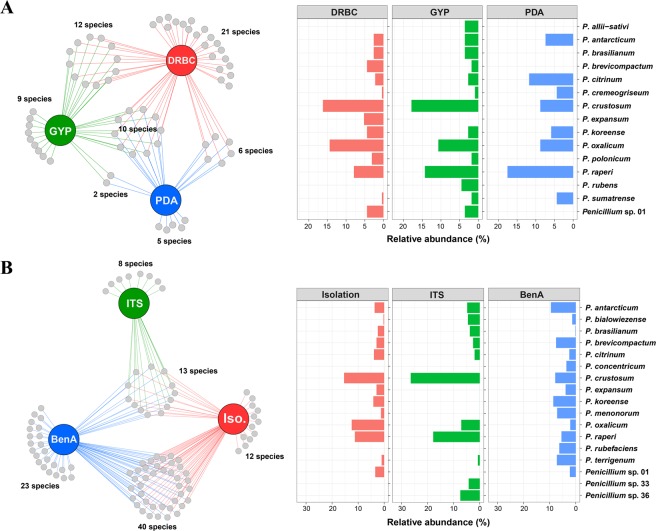
*Penicillium* diversity detected from intertidal zone. (**A**) Total, unique, and shared *Penicillium* species and relative abundance of major species (3%) from three different media (DRBC, GYP, and PDA). (**B**) Total, unique, and shared *Penicillium* species and relative abundance of major species (3%) obtained from isolation, NGS using ITS, and NGS using *benA*.

Different numbers of *Penicillium* species were isolated depending on the isolation medium: 49 species were isolated on DRBC, 33 species on GYP, and 23 species on PDA (Table S1). Ten species were isolated on all media. A total of 21, 9, and 5 species were exclusively isolated on DRBC, GYP, and PDA, respectively (Fig. 2A). *Penicillium crustosum* was the predominant species, followed by *P*. *oxalicum* and *P*. *raperi*.

### *Penicillium* diversity from culture-independent approach

For ITS-NGS, a total of 2,363,934 fungal sequence reads were obtained, of which 1,813,137 reads were retained after filtering low quality sequences. Most Good’s coverage estimates were 0.955–0.997, indicating a sufficient coverage for diversity analysis. One sample had a score of 0.890. Among the total number of sequences, *Penicillium* sequences accounted for 1,301 reads, and 21 mOTUs (99% threshold) were assigned to 21 species based on the phylogenetic analysis (Table S2 and Fig. S1). The mOTUs were identified to the species level based on a section-by-section phylogenetic analysis using ITS sequences. A total of 13 species were identified to known *Penicillium* species, and the remaining eight species could not be confidently identified because of unclear phylogenetic relationships. *Penicillium crustosum* (27.8%) was the predominant species, followed by *P*. *raperi* (18.7%), *Penicillium* sp. 36 (7.8%), and *P*. *oxalicum* (7.4%) (Fig. 2B).

For *BenA-*NGS, a total of 1,513,345 fungal sequence reads were obtained, of which 860,499 reads were retained after filtering the low quality and non-*Penicillium* sequences. A total of 970 mOTUs (99% threshold) were detected, representing 76 species based on the phylogenetic analysis (Table S2 and Fig. S2). All Good’s coverage estimates were 0.989–1.000, indicating that sequencing depth was appropriate for the representing *Penicillium* diversity. *Penicillium antarcticum* (9.5%) was the predominant species, followed by *P*. *koreense* (8.6%), *P*. *crustosum* (7.9%), *P*. *brevicompactum* (7.6%), *P*. *terrigenum* (7.3%), *P*. *menonorum* (7.2%), *P*. *rubefaciens* (6.3%), and *P*. *raperi* (5.5%) (Fig. 2B).

### Comparison of culture-dependent and –independent approaches

In total, 96 *Penicillium* species were detected from mudflat and sand using the three different methods (conventional isolation, ITS-NGS, and *BenA*-NGS) (Fig. 2B and Table S2). Each method identified a different number of species: 65 species from isolation, 21 species from ITS-NGS, and 76 species from *BenA*-NGS. A total of 13 species were shared across all three methods. Particularly, *P*. *antarcticum*, *P*. *crustosum*, and *P*. *raperi* were commonly detected from mudflat and sand in all methods. Several species were detected by only one method: isolation (12 species), ITS-NGS (8 species), and *BenA*-NGS (23 species) (Fig. 2B). *Penicillium rubefaciens* and *P*. *concentricum* were commonly detected in *BenA*-NGS, but not detected in isolation and ITS-NGS.

### Community structure of *Penicillium*

**Figure 3:**
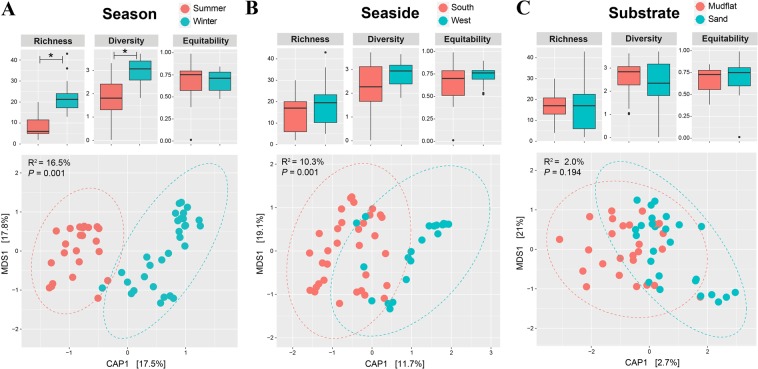
Alpha diversity and Constrained Analysis of Principal coordinates (CAP) plots for *Penicillium* communities in season, seaside, and substrate. Significance of difference in diversity indices was tested by Wilcox rank sum test with FDR correction (*P < 0.05). CAP plots for *Penicillium* communities based on Bray-Curtis distance were constrained by (**A**) season, (**B**) seaside, or (**C**) substrate. Significance of CAP models was evaluated using ANOVA with 999 permutations.

Diversity and community analyses were conducted based on the *BenA*-NGS dataset because it represents *Penicillium* communities the best, with exact species-level identification and high coverage power for detecting *Penicillium* species compared to other methods. Alpha diversity indices for *Penicillium* communities were compared between season (summer and winter), seaside (western and southern coast), and substrate (mudflat and sand) (Fig. 3). Species richness and diversity were significantly different based on season (Richness: P < 0.001; Diversity: P < 0.001), while evenness was comparable (P = 0.702). The winter season had a higher richness and diversity compared to the summer season. However, no index showed a significant difference for seaside (Richness: P = 0.189; Diversity: P = 0.164; Equitability: P = 0.180) and substrate (Richness: P = 0.634; Diversity: P = 0.634; Equitability: P = 0.634).

**Figure 4:**
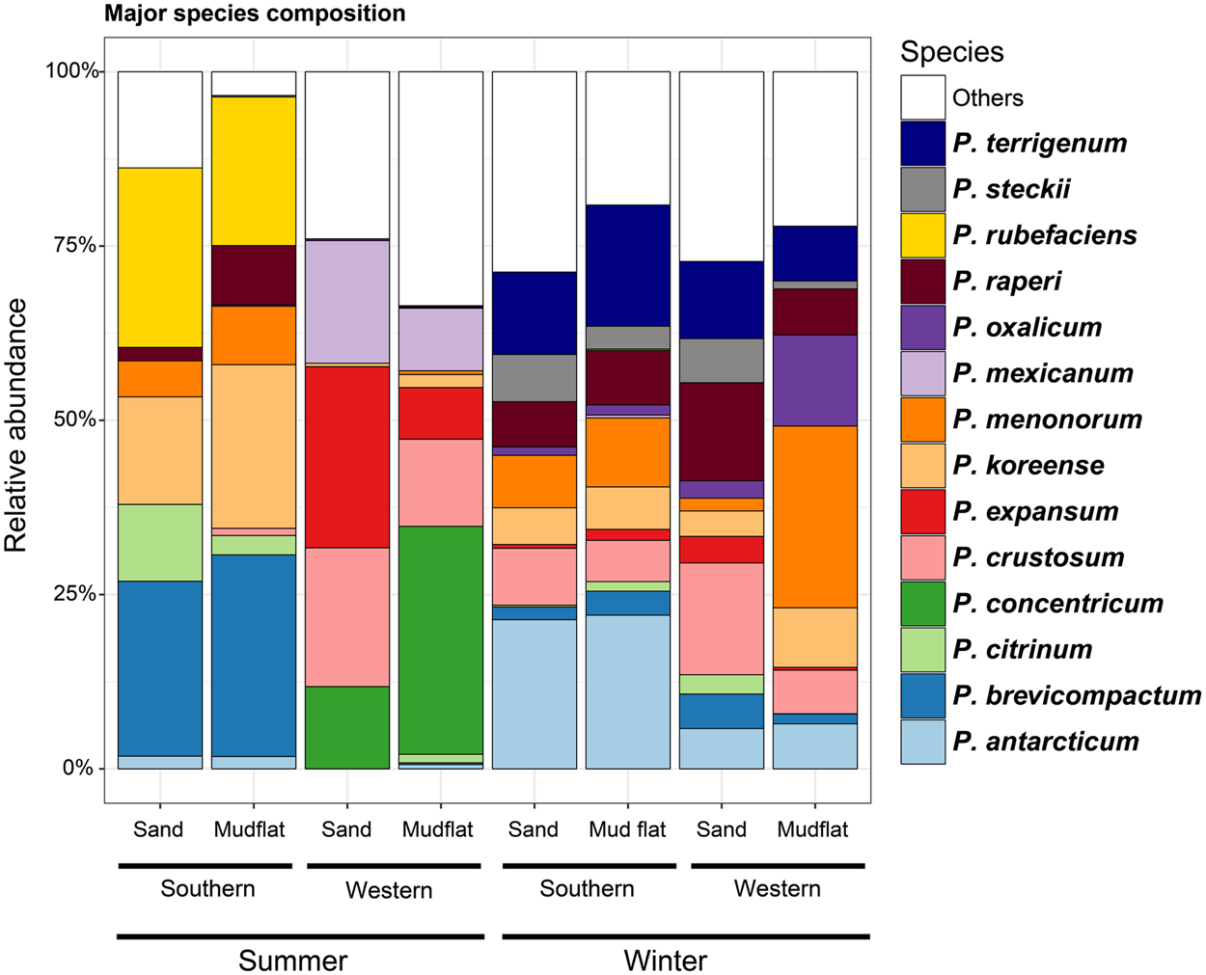
Composition of major *Penicillium* species obtained from NGS using *benA*. Relative abundance of major species obtained in season (summer and winter), seaside (western and southern coast), and substrate (mudflat and sand) from South Korea.

*Penicillium* communities were compared between season, seaside, and substrate using CAP analysis (Fig. 3). The community structure of *Penicillium* was significantly different depending on season (P = 0.001, 16.5% explanatory power) (Fig. 3A). *Penicillium brevicompactum*, *P*. *concentricum*, *P*. *expansum*, *P*. *koreense*, *P*. *mexicanum*, and *P*. *rubefaciens* were more abundant in the summer than the winter (Fig. 4). In contrast, *P*. *antarcticum* and *P*. *terrigenum* were more abundant in the winter. Seaside had a significant effect on the clustering of communities (P = 0.001, 10.3% explanatory power) (Fig. 3B). Among major species, *P*. *antarcticum*, *P*. *brevicompactum*, *P*. *koreense*, *P*. *rubefaciens*, and *P*. *terrigenum* were significantly more abundant from the southern coast compared to the western coast (Fig. 4). In contrast, *P*. *concentricum*, *P*. *expansum*, and *P*. *mexicanum* were significantly more abundant on the western coast. Substrate did not significantly influence the *Penicillium* community structures (P = 0.194, 2.0% explanatory power) (Fig. 3C).

### Halotolerance, enzyme activity, and PAH degradation

**Figure 5:**
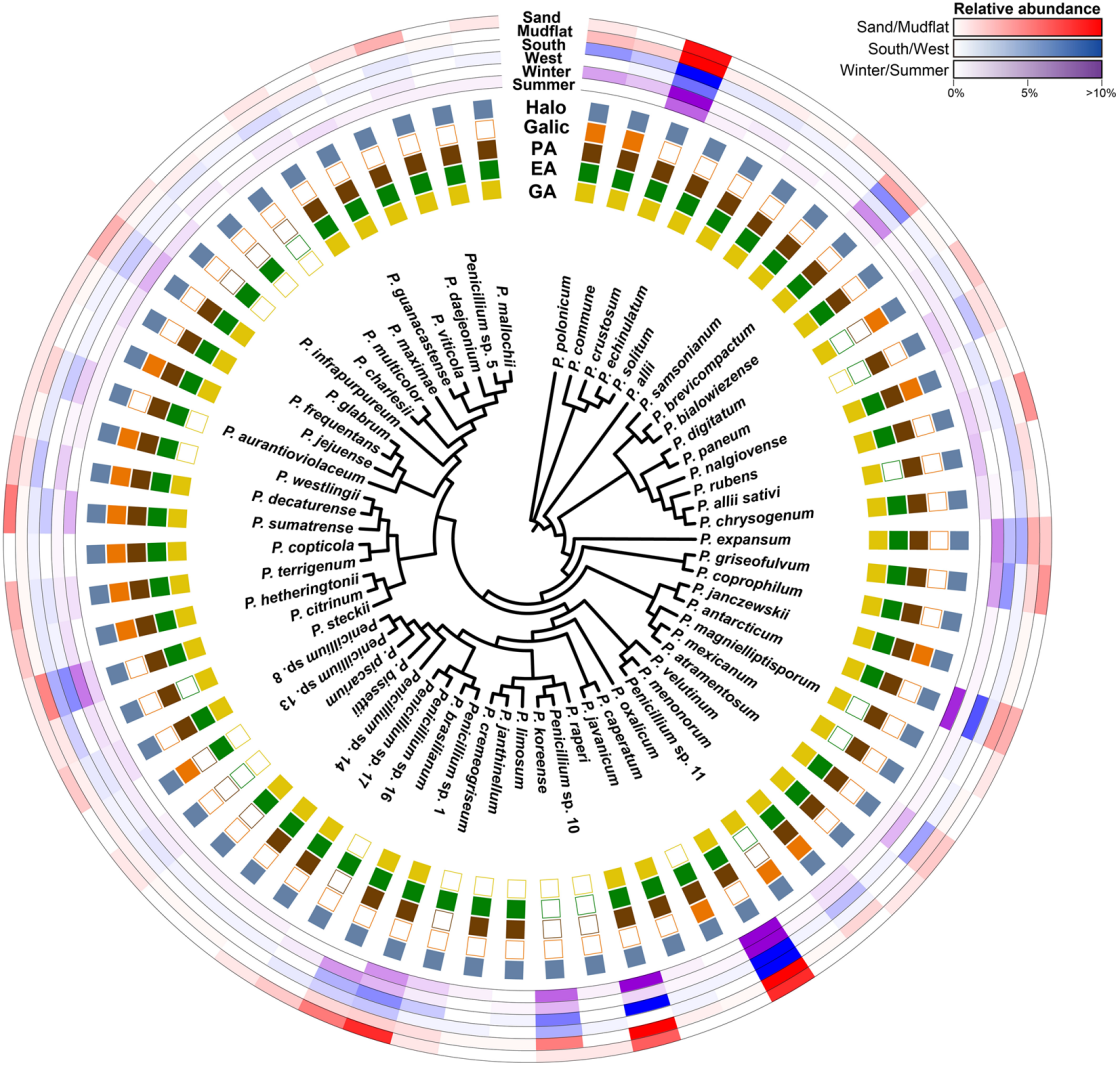
The relative abundance and physiological characteristics (halotolerance, enzyme activity, and polycyclic aromatic hydrocarbons [PAHs] degradation) of the *Penicillium* strains in a phylogenetic context. The neighbor-joining phylogenetic tree was based on partial *BenA* gene sequences. The relative abundance of *Penicillium* isolated from intertidal zone is color coded on the outer ring (substrate – red; season – blue; seaside – purple). Halotolerance, enzyme activity, and PAH degradation of the *Penicillium* is color coded on the inner ring (Halotolerance – light blue; gallic acid reaction – orange; protease – brown; endoglucanase – green; β-glucosidase - yellow).

All species recovered from isolation showed halotolerance (Fig. 5). Among the 65 species, 54 species showed β-glucosidase activity, with *P*. *citrinum* (SFC20151118-M02) and *P*. *raperi* (SFC20150915-M08) exhibiting the strongest activity. Fifty species showed endoglucanase activity, with *P*. *bialowiezense* (SFC20151014-M03), *P*. *rubens* (SFC20151014-M07), and *P*. *hetheringtonii* (SFC20151014-M08) exhibiting relatively strong endoglucanase activity. Among 53 species showing protease activity, *P*. *guanacastense* (SFC100711) and *Penicillium* sp. 5 (SFC100716) showed the strongest activity. For PAH degradation, 16 species showed a positive reaction as indicated by a brown color. *Penicillium caperatum* (SFC20151118-M06), *P*. *decaturense* (SFC20150303-M14), *P*. *hetheringtonii* (SFC20151014-M08), and *P*. *janczewskii* (SFC20151014-M10) showed the strongest positive reaction (Table S1).

## Discussion

### *Penicillium* diversity using culture-dependent and –independent approaches

We conducted a comphrensive study to determine the species richness of *Penicillium* found in the intertidal zone along the Korean coast, testing for impact of season, seaside, and substrate. A total of 96 *Pencillium* species were identified using three different methods (isolation, ITS-NGS, *BenA*-NGS). We identified 54 more species than the number of *Penicillium* species previously recorded in Korea^27^ and 58 more species than the number of *Penicillium* species reported from the marine environment globally^4–6,28–31^. Most of the previous studies of marine *Penicillium* from intertidal zones focused on screening for industrially useful enzymes as well as novel bioactive compounds. Therefore, these studies primarily identified strains to the genus level only and a handful to the species level (*Penicillium chrysogenum*, *Penicillium citrinum*) using ITS sequences^32,33^. Compared to our previous studies^5,34^, we identified 41 species new to intertidal zones, as well as 17 new species candidates.

Most previous studies used either a culture-dependent method^35,36^ or a culture-independent method using a single locus (e.g. ITS)^37,38^. We employed three methods to detect as many *Penicillium* species as possible and to compare the effectiveness of the three methods. Generally, a culture-dependent method isolates only a small subset of the microbial diversity from environments^39^. In our study, a relatively high proportion of *Penicillium* species (63.1%) were detected using a culture-dependent method. This is likely due to the fact that we focused on *Penicillium*, which are generally easy to culture; many *Penicillium* species are isolated from various substrates from terrestrial environments^1,2,40,41^ and marine environments^4–7^. Another reason for the high detection is that we used three different media for isolation. More *Penicillium* species were isolated from the DRBC medium compared to the other two media. DRBC medium suppresses fast growing fungi, while the other isolation media do not^42^. Some *Penicillium* species grow slowly and may be covered by fast growing fungi^43^. Several species were only isolated on one agar medium (Fig. 2A), but there was no apparent tendency for them to prefer certain media. Therefore, more different media have the opportunity to isolate the more diverse *Penicillium* species.

Although NGS methods help to detect more diverse fungi within the environment, molecular marker selection has a significant impact on NGS methods. The ITS region is less discriminative in identifying species of *Penicillium* than *BenA*. However, we use ITS, as well as *BenA*, since it is widely accepted fungal barcode^22^. In our study, certain species have been detected only in ITS-NGS. Among the 84 species detected in the NGS analysis, 15.5% of the *Penicillium* species were shared between ITS-NGS and *BenA*-NGS, while 9.5% were exclusive to ITS-NGS and 75.0% exclusive to *BenA*-NGS. A total of 20 *Penicillium* species were not detected in *BenA*-NGS (8 species from ITS-NGS and 12 species from isolation). The phenomenon of mismatched fungal diversity depending on the survey methods has been seen in previous studies^44–47^, which can result from the technical bias associated with DNA extraction, PCR amplification, and sequencing^48^. Low abundance species were generally prone to technical bias, which agrees with our results (Table S2). In the case of ITS-NGS, low sequence variation of the ITS2 region can be one of the sources of diversity difference to *BenA*-NGS; some *Penicillium* species cannot be assigned to species level based on the ITS2 region. If we consider the potential species based on ITS2 similarity (Table S2 and Fig. S1), five more species can be added to the number of shared mOTUs (*Penicillium* sp. 33, 34, 36, 38, 39). The unique species detected using the isolation method may correspond to the species occurring at low concentration; the culture-dependent method has been shown to detect bacteria at a broader range of concentrations that is 10^2^–10^4^ times lower than that of the culture-independent method^49^. The higher number of mOTU detected by *BenA*-NGS compared to ITS-NGS suggests that it is useful to develop genus-specific primers for protein-coding genes (compared to ITS) for species identification and species richness studies. This is the first study using *BenA*-NGS for *Penicillium* diversity, and demonstrates its usefulness for detecting *Penicillium* in the environment. However, as with using multiple media for culture studies, combining multiple molecular markers will likely identify a greater species richness.

### *Penicillium* community structure depends on environmental factors

We found that *Penicillium* diversity was significantly higher in winter than summer. Given that the diversity did not differ between substrate and seaside, seasonal variation seems to be an important factor influencing *Penicillium* diversity. High diversity in winter was also detected in studies of terrestrial fungi^50,51^, which may relate to severe environmental conditions. Various species can co-exist together in harsh environments, and, when conditions are relaxed, a single or small number of species are able to dominate and exclude other species^50^. In the winter season, *Penicillium* may be in spore state or grow slowly, which prevents the dominance of a single species. On the other hand, nutrient deficiency may occur in winter. Since coastal zones are influenced by river flows and oceanic currents, nutrient supply can fluctuate seasonally; nutrient supply from rivers is high in summer and low in winter^52,53^. Oligotrophic condition in winter may lead to co-existence of various *Penicillium* species.

There were different patterns of the *Penicillium* community between the western and southern coasts. The difference in *Penicillium* community between seasides is likely influenced by the difference in oceanic currents in each region. The western coast is influenced by the cold currents of West Korea Coastal Current during winter and summer, whereas the southern seaside is influenced by both the Jeju Warm Current and Taiwan Warm Current during winter and summer^54^. Ocean current patterns influence on the community structure of bacteria, ammonia-oxidizing archaea, and animal biodiversity^55–57^. Previous studies showed that temperature influences fungal community in marine environments^58,59^. The ocean currents likely provide temperature and physical barriers limiting *Penicillium* dispersal and affect *Penicillium* diversity. Some marine fungi have shown the differential preference on the plant species^60,61^, thus the vegetation composition in the coasts can be another environmental factor that influences on the fungal community structure. In South Korea, the flora of western and southern coasts is different^62,63^, which likely influences the *Penicillium* communities.

Considering physiochemical differences between mudflat and sand (*e*.*g*. texture and nutrient composition), different *Penicillium* communities were expected. However, the substrate did not significantly influence the diversity and community structure of *Penicillium* (Fig. 3C). Though environmental filtering by substrates is important factor to differentiate fungal communities^64–67^, marine fungal community structures usually depend on the biological substrate such as animal, algae, or plant^15,35,68^. Macroalgae, sponge, and animal debris have been known to harbor high *Penicillium* diversity^5,7,69,70^. High enzyme activity associated with alginate, cellulose, and protein detected in *Penicillium* isolates supports this speculation.

### Ecological roles of *Penicillium* associated with enzyme activities

*Penicillium* species from various environments produce a variety of enzymes such as alginase, cellulase, chitinase, and proteasee^5,7,71,72^. Protease activity has been reported in fungi isolated from various marine environments^73,74^. Particularly, members in *Penicillium* are known for their biotechnological potential in the production of proteases^72^. In our study, a relatively high proportion of *Penicillium* from the intertidal zone exhibited endoglucanase, β-glucosidase, and protease activity. A high proportion of species having enzyme activities and halotolerance implies that these species play an important role as decomposers of cellulose and protein in the intertidal zone. Macroalgae (over 400 species reported to date)^75^ and animal debris such as crab, fish, shells, and sponges are frequently found in the intertidal zone. *Penicillium* species that have a high enzyme activity can use this abundant algae and animal debris on the intertidal zone as a their favorite nutrient source. Although the species in the *Penicillium* sections *Brevicompacta*, *Citrina*, *Canescentia*, *Fasciculata*, and *Sclerotiora* showed relatively higher enzyme activity than the species in the other sections, there was no distinct pattern of enzyme activity based on a phylogenetic group.

Microbes are known to degrade various pollutants that result from human activities in intertidal zones^76^. Members in the genus *Penicillium* have been shown to degrade environmental pollutants such as synthetic dyes and polycyclic aromatic hydrocarbons (PAHs)^72^. PAHs are produced by the incomplete combustion of organic matter such as oil and wood^77^. *Penicillium canescens*, *P*. *chrysogenum*, *P*. *frequentans*, *P*. *italicum*, *P*. *janthinellum*, *P*. *montanense*, *P*. *simplicissimum*, and *P*. *restrictum* are known to degrade PAHs^78^, but these species did not show PAHs degrading ability in our study. The PAHs-degrading ability of fungi varies by strain and species. The capability of PAHs degradation was observed in only a few *Penicillium* species in this study. Particularly, the species (*P*. *decaturense* and *P*. *hetheringtonii*) in section *Citrina* showed a relatively higher ability to degrade the PAHs compared to species in other sections.

## Conclusions

The number of studies investigating marine fungi has increased in recent years and *Penicillium* are one of the many fungi reported from this environment. In this study, we detected 96 *Penicillium* species from mudflat and sand from Korea using three different analysis techniques (conventional isolation, ITS-NGS, and *BenA*-NGS). Although *BenA*-NGS detected the highest number of *Penicillium* species, some *Penicillium* species were only detected by isolation and/or ITS-NGS method. If the goal is to identify the total species diversity, we suggest combining various approaches to detect more species. Many of the isolated *Penicillium* exhibited cellulase activity, protease activity, and/or degradation of PAHs. *Penicillium* is a decomposer of a variety of marine organisms and it is thought to play an important role in nutrient recycling and pollutant degradation in intertidal environments. However, it is unclear whether the Penicillia identified in this study are in an active or inactive form in the marine environment. An additional study will be needed to further identify what is the actual function of the Penicillia in the marine environment. We expect that this study will provide basic information regarding the *Penicillium* diversity and improve interest in the functional role of Penicillia in marine environments.

## Materials and Methods

### Sample collections

During summer (July and August in 2014) and winter (February 2015), mudflat and sand samples were collected from 30 coastal sites around Korea (10 western coast, 20 southern coast) for each season (Fig. 1). Samples were collected during low tide. To fully characterize each site, each sample contained five subsamples collected approximately 20 m from each other. Each subsample was collected at 2–3 cm depth after removing the top soil to avoid surface contamination. Samples were transported on ice and stored at 4 °C for isolation and at −80 °C for DNA extraction.

### Diversity analysis by culture-dependent approach

#### Isolation of fungi

For each sample, 5 g was tenfold diluted with sterilized artificial sea water (ASW)^79^. For each dilution, 100 μL was plated on potato dextrose agar (PDA, Difco, Becton Dickinson, Sparks, MD, USA), glucose yeast extract agar (GYP; 1 g L^−1^ glucose, 0.1 g L^−1^ yeast extract, 0.5 g L^−1^ peptone, and 15 g L^−1^ agar), and dichloran rose bengal chloramphenicol agar (DRBC; Difco, Becton Dickinson, Sparks, MD, USA). We performed a preliminary test for screening the growth of *Penicilium* at different temperatures (10 °C, 15 °C, 20 °C, 25 °C, 30 °C) on malt extract agar (MEA; Oxoid) and chose 25 °C for incubation since most species grew well at 20–30 °C (Table S3). In particular, several species (*P*. *brasilianum*, *P*. *decaturense*, *P*. *griseofulvum*, *P*. *solitum*, and *Penicillium* sp. 13 were detected only during winter, but showed optimal growth at 25–30 °C. All plates were incubated at 25 °C for 7–14 days. We transferred all *Penicillium* to a new PDA plate. The isolated strains were stored in 20% glycerol at −80 °C at the Seoul National University Fungus Collection (SFC) (Table S1).

#### DNA extraction, PCR amplification, and sequencing from isolates

Genomic DNA was extracted from isolated *Penicillium* strains using the modified cetyltrimethylammonium bromide (CTAB) extraction protocol^80^. The PCR amplifications of the ITS and *BenA* region were performed using primers ITS1F/ITS4^81^ and Bt2a/Bt2b^82^, respectively. Each PCR reaction was performed in a C1000 thermal cycler (Bio-Rad, Richmond, CA, USA) following previously described methods^5^. The PCR products were purified using the Expin^TM^ PCR Purification Kit (GeneAll Biotechnology, Seoul, Korea) according to the manufacturer’s instructions. Sequencing was performed in both forward and reverse directions using the corresponding PCR primers at Macrogen (Seoul, Korea), using an ABI Prism 3730 genetic analyzer (Life Technologies, Gaithersburg, MD, USA).

#### Identification of *Penicillium* isolates

Sequences were assembled, proofread, edited, and aligned using MEGA5^83^. Consensus sequences were deposited in GenBank (Table S1). Molecular identification of species was performed in two steps. First, the sectional position of the strains was determined by comparison of the ITS sequences in a dataset containing sequences of type strains. Next, the *BenA* sequence data were compared with type strain sequences to identify strains at the species level. Multiple alignments were performed using MAFFT v. 7^84^ and the L-INS-I algorithm. Neighbor joining (NJ) trees were constructed with MEGA 5 using Kimura 2-parameter model^85^ with 1,000 bootstrap replicates.

### Diversity analysis by culture-independent approach

#### DNA extraction, PCR amplification, and sequencing from environmental DNA (eDNA)

For the culture-independent approach, eDNA was extracted directly from 500 mg of substrate samples (mudflat or sand) using the MoBio PowerSoil DNA isolation kit (MoBio laboratories, Carlsbad, CA, USA) following the manufacturer’s instructions. Each PCR reaction was performed in a C1000 thermal cycler (Bio-Rad) using AccuPower® PCR PreMix (Bioneer) in a final volume of 20 μL, containing 10 pmol of each primer and 1 μl of DNA. For ITS, PCR amplification was performed using the primers ITS3 and ITS4^81^ attached with Illumina sequencing adaptors. The PCR program was as follows: 5 min at 94 °C; 35 cycles of 30 s at 94 °C, 30 s at 55 °C, and 40 s at 72 °C; and a final extension step of 5 min at 72 °C. For *BenA* amplification, we designed *Penicillium* specific primers: forward primers BenA-F (ATCGGTGCTGCTTTCTGGTA) and BenA-2F (CGGTGCTGCTTTCTGGTANGT); reverse primers BenA-2R (TGRCCGAARACRAARTTRTCGG) and BenA 300R (GCRTCCATRGTRCCRGGYTC).

The first PCR was performed using primers BenA-F and BenA-2R, followed by a second PCR using primers BenA-2F and BenA 300 R attached with Illumina sequencing adaptors. The program of the first PCR was as follows: 5 min at 94 °C; 35 cycles of 30 s at 94 °C, 30 s at 58 °C, and 40 s at 72 °C; and a final extension step of 5 min at 72 °C; the second PCR program was 5 min at 94 °C; 35 cycles of 30 s at 94 °C, 30 s at 68 °C, and 40 s at 72 °C; and a final extension step of 5 min at 72 °C. PCR products were confirmed using gel electrophoresis and purified using the Expin^TM^ PCR Purification Kit (GeneAll Biotechnology, Seoul, Korea) according to the manufacturer’s instructions. Individual PCR products were quantified using a NanoDrop 2000 spectrophotometer (Thermo, USA) and a total of 300 libraries were pooled. Sequencing was performed with the Illumina MiSeq system (Illumina) at Macrogen (Seoul, Korea). Paired-end sequences were generated and deposited at NCBI Sequence Read Archive (PRJNA529042).

#### Bioinformatics process and statistical analysis

The Illumina MiSeq generated ITS (ITS-NGS) and *BenA* (*BenA*-NGS) sequence data sets were processed using Quantitative Insights Into Microbial Ecology (QIIME) v. 1.8.0^86^. Sequence pairs were assembled using fastq-join and low quality (QV < 20), short (<200 bp) sequences filtered during the de-multiplexing process. Molecular operational taxonomic units (mOTUs) were clustered with 99% similarity threshold and chimera sequences were filtered using *de novo* clustering in USEARCH 5.2.236^87^. For each mOTU, the most abundant sequence was chosen as the representative sequence and used for pre-identification using BLAST against type sequences of *Penicillium* for ITS and *BenA*. Final identification was conducted based on phylogenetic analysis as described above. Additional chimeric sequences were filtered based on each database using by UCHIME v4.2^88^. Non-fungal, non-*Penicillium* sequences, and mOTU with less than 10 reads were removed from the analysis. Before the next step, sequences were rarified based on the minimum number of sequences for normalizing dataset. Indices of alpha diversity were calculated for richness (Chao1), diversity and evenness (Shannon’s index) in QIIME.

Statistical tests and graphical plotting were conducted using ggplot2^89^, phyloseq^90^, and basic packages in R^91^. Alpha diversities were compared between categories (season, seaside, and substrate) using Wilcox rank sum test adjusted by the false discovery rate (FDR)^92^. Community structures were compared by Constrained Analysis of Principal coordinates (CAP) based on Bray-Curtis dissimilarities. CAP models were constrained by each category with conditioning by the other factors (e.g. ~Seaside + Condition [Season + Source]), and significance was tested by ANOVA-like tests with 999 permutations.

### Halotolerance, enzyme activity, and PAH degradation

Halotolerance was determined by measuring the colony diameter of a representative strain of each species on MEA supplemented with ASW instead of distilled water. Each species was inoculated in three-point fashion on MEA with and without ASW using spore suspensions. The plates were incubated at 25 °C and colony diameter was measured after 5 days. Growth difference between MEA with and without ASW was compared.

Endoglucanase, β-glucosidase, and protease activity were assessed for a representative strain of each species using a modified plate screening method^69^. Endoglucanase and β-glucosidase activity were assayed on Mandels’ medium^71^, with 1% carboxymethylcellulose (Sigma-Aldrich, MO, USA) and 0.5% D-cellobiose (Sigma-Aldrich, MO, USA) as the primary carbon source, respectively. Protease activity was assayed by growing the fungi for five days on yeast extract agar (Oxoid, MD, USA) supplemented with 1.5% skim milk (Difco-Becton, MD, USA) as the primary carbon source^93^. To indicate the enzyme activity, the diameters (mm) of clear zones surrounding colonies were measured.

A gallic acid reaction was used as a screening method to determine the capability of degradation of PAHs^94^. The gallic acid reaction was assayed by growing the fungi on 1.5% MEA (Difco-Becton, MD, USA) supplemented with 5 g L^−1^ of gallic acid. Each plate was incubated at 25 °C for 14 days. The capability of degradation of PAHs was identified by a color change surrounding the colony. Each value for a strain is the average result from three experiments.

## Supplementary information

Supplementary information
