# Supplementary material for: The diversity and ecological roles of Penicillium in intertidal zones
Source: Sci Rep. 2019 Sep 19;9:13540. doi: 10.1038/s41598-019-49966-5 (PMC6753150; doi:10.1038/s41598-019-49966-5)
Supplement: Supplementary file 1 — Supplementary information [file 41598_2019_49966_MOESM1_ESM.pdf]

Supplementary Information

The diversity and ecological roles of *Penicillium* in intertidal zones

Myung Soo Park[1], Seung-Yoon Oh[1], Jonathan J. Fong[2], Jos Houbraken[3], Young Woon Lim[1]*

[1]School of Biological Sciences and Institute of Microbiology, Seoul National University, Seoul 08826, South Korea

[2] Science Unit, Lingnan University, Tuen Mun, Hong Kong

[3]Westerdijk Fungal Biodiversity Centre, Uppsalalaan 8, 3584 CT Utrecht, Netherlands

Supplementary Table 1 Information, halo-tolerance, enzyme activity, gallic acid reaction, GenBank accession numbers for *Penicillium* species isolated from sand and mudflat along the west and south coast of South Korea

Species	Strain	Number of strain			Halo-tolerance[a]	Clear zone (mm)[b]			Gallic acid reaction	Accession No. (*BenA*)
		DRBC	GYP	PDA		EA	GA	PA		
P. allii	SFC20151118-M09	2			T	4.5	1	9.5	-	MK682830
P. allii-sativi	SFC20151118-M13		8		T	2.5	0	6	-	MK682831
P. antarcticum	SFC20150915-M01	12	8	10	T	1.5	3.5	10.5	-	MK682832
P. atramentosum	SFC20150402-M34	10	2	4	T	2.5	2	3	-	MK682833
P. aurantioviolaceum	SFC20150303-M06	10		2	T	0	4	3	-	MK682834

P. bialowiezense	SFC20151014-M03	2			T	7	6.5	11	-	MK682835
P. bissettii	SFC100822			2	T	3.5	1	0	-	MK682836
P. brasilianum	SFC20150303-M10	12	8		T	2.5	1	1	-	MK682837
P. brevicompactum	SFC20151014-M11	20	4		T	5	6.5	12.5	-	MK682838
P. caperatum	SFC20151118-M06	2			T	0	2	3	+	MK682839
P. charlesii	SFC20150915-M13	4			T	0	1	0	-	MK682840
P. chrysogenum	SFC20151014-M02		6		T	1	6	5	-	MK682841
P. citrinum	SFC20151118-M02	10	6	16	T	5	9	8	-	MK682842
P. commune	SFC20151118-M04	10	2		T	1	6	4	+	MK682843
P. coprophilum	SFC20150303-M11	2			T	5	6	9	-	MK682844
P. copticola	SFC20150303-M12		2		T	3.5	6	9.5	+	MK682845
P. cremeogriseum	SFC20151014-M15	2	2	6	T	0	1	0	-	MK682846
P. crustosum	SFC20151118-M08	74	40	12	T	2.5	5.5	2	-	MK682847
P. daejeonium	SFC20151014-M05		2	4	T	3	3	12	-	MK682848
P. decaturense	SFC20150303-M14	6	4		T	3	2.5	5.5	+	MK682849
P. digitatum	SFC20151014-M04	4			T	2	0	0	+	MK682850
P. echinulatum	SFC20151014-M09	4			T	3	6	6.5	-	MK682851
P. expansum	SFC20150402-M38	24			T	3	4	8.5	-	MK682852
Penicillium sp. 05	SFC100716			2	T	1	6	13	-	MK682887
P. frequentans	SFC20150915-M09	2		2	T	1.5	2	5	-	MK682853
P. glabrum	SFC20150915-M02	10	4		T	2	5	12	-	MK682854
P. griseofulvum	SFC20151118-M01	12	6		T	3	6	2.5	-	MK682855

P. guanacastense	SFC100711		4		T	3.75	6	13	-	MK682856
P. hetheringtonii	SFC20151014-M08	4	2		T	6	5	8	+	MK682857
P. infrapurpureum	SFC20150915-M12	2		2	T	0	2	0	-	MK682858
P. janczewskii	SFC20151014-M10		2		T	5	5.5	10	+	MK682859
P. janthinellum	SFC20151118-M12	2			T	0	4	1	-	MK682860
P. javanicum	SFC20151118-M07	2		2	T	5.5	4	2	-	MK682861
P. jejuense	SFC20150402-M41	2			T	2	1.5	10	+	MK682862
P. koreense	SFC20151014-M13	20	6	8	T	0	0	0	-	MK682863
P. limosum	SFC20151118-M05	2			T	0	2	3.5	-	MK682864
P. magnielliptisporum	SFC20151118-M03		2		T	3	0	2	-	MK682865
P. mallochii	SFC20150915-M03	2			T	2.5	5	12	-	MK682866
P. maximae	SFC20151014-M14	6			T	1	5	10	-	MK682867
P. menonorum	SFC20150915-M07	6		4	T	4.5	5	1	+	MK682868
P. mexicanum	SFC20151014-M01		2		T	1	0	1	-	MK682869
P. multicolor	SFC20151118-M10	2			T	0	0	0	-	MK682870
P. nalgiovense	SFC20150402-M42			4	T	3	5	1	+	MK682871
P. oxalicum	SFC20150915-M10	66	24	12	T	2	2	12.5	-	MK682872
P. paneum	SFC20150915-M14	6		4	T	0	0	1	-	MK682873
P. piscarium	SFC20151014-M16	2	4		T	0	0	0	-	MK682874
P. polonicum	SFC20151014-M06	14	4		T	4	6	11.5	+	MK682875
P. raperi	SFC20150915-M08	36	32	24	T	1.5	8	2	-	MK682876
P. rubens	SFC20151014-M07		10		T	6	6	1	-	MK682877

P. samsonianum	SFC100899	2			T	4.5	5	4	-	MK682878
P. solitum	SFC100989		4		T	2	2	5	-	MK682879
P. steckii	SFC20150303-M17	4	2	2	T	4.5	0	10	-	MK682880
P. sumatrense	SFC20150915-M04	2	4	6	T	1	2	5.5	+	MK682881
P. terrigenum	SFC20150915-M06		4	4	T	4.5	7	6	+	MK682882
P. velutinum	SFC20150915-M11			2	T	1.5	4.5	2.5	-	MK682883
P. viticola	SFC20150915-M05	2			T	2	4	12.5	-	MK682884
P. westlingii	SFC20151118-M11	4			T	0	4	6	+	MK682885
Penicillium sp. 01	SFC20150303-M09	20	8		T	1	1	2.5	-	MK682886
Penicillium sp. 08	SFC100917	4	4		T	1	2	1	-	MK682888
Penicillium sp. 10	SFC100725			2	T	0	0	0	-	MK682889
Penicillium sp. 11	SFC101038	2			T	4	0	0	+	MK682890
Penicillium sp. 13	SFC100985	4			T	0	1	0	+	MK682891
Penicillium sp. 14	SFC100817	2			T	3	7	1.5	-	MK682892
Penicillium sp. 16	SFC100919	2	2		T	0	1	0	-	MK682893
Penicillium sp. 17	SFC100747	2			T	1	2	1	-	MK682894

[A]Species showed the best growth on MEA supplemented with artificial sea water (ASW) and without ASW (Corrected $P < 0.05$) are represented as “T”

[b]Endoglucanase (EA), β-glucosidase (GA), and protease (PA) activity

Supplementary Table 2 The number of strains or reads of *Penicillium* species detected from substrate (sand and mudflat), season (summer and winter), and seaside (west and south) using isolation (C), NGS using ITS (IN), and NGS using *benA* (BN)

Species	Seaside						Season						Substrate						Totals		
	West			South			Summer			Winter			Sand			Mudflat					
	C	IN	BN	C	IN	BN	C	IN	BN	C	IN	BN	C	IN	BN	C	IN	BN	C	IN	BN
P. adametzii		2	154			29		1			1	183		2	10			173		2	183
P. allii				2			2									2			2		
P. allii-sativi				8			8									8			8		
P. antarcticum	26	57	11151	4	9	70944	28	60	4897	2	6	77198	6	14	41925	24	52	40170	30	66	82095
P. atramentosum			10	16			2			14		10	4		10	12			16		10
P. aurantioviolaceum	8		1319	4		2	2			10		1321	2		109	10		1212	12		1321
P. bialowiezense		37	3078	2	25	8571		41	2827	2	21	8822		35	328	2	27	11321	2	62	11649
P. bissettii	2		127			15973	2		21			16079			1568	2		14532	2		16100
P. brasilianum	12	48	174	8	4	1057		47		20	5	1231	10	27	1018	10	25	213	20	52	1231
P. brevicompactum	4	8	5837	20	27	59258	2	18	50558	22	17	14537	2	4	33312	22	31	31783	24	35	65095
P. cairnsense			266						266									266			266
P. caperatum	2		110							2		110				2		110	2		110
P. charlesii			62	4						4		62			61	4		1	4		62
P. chrysogenum			2589	6		476	4			2		3065			2013	6		1052	6		3065
P. citrinum	18	3	3829	14	24	17473	6	21	15963	26	6	5339	2	26	11271	30	1	10031	32	27	21302
P. commune	2		296	10		101				12		397			332	12		65	12		397
P. concentricum			30920			17			30919			18			9924			21013			30937
P. coprophilum			38	2						2		38			38	2			2		38
P. copticola				2						2						2			2		
P. corylophilum			4027			3691			4027			3691			3800			3918			7718
P. cremeogriseum	8		3122	2		859	6		2886	4		1095	2		847	8		3134	10		3981

P. crustosum	22	220	44774	104	142	23213	20	208	25365	106	154	42622	18	206	29461	108	156	38526	126	362	67987
P. daejeonium	2	19	7023	4	1	7496	2	17	1250	4	3	13269	6	15	12769		5	1750	6	20	14519
P. daleae			3403			676			1644			2435			16			4063			4079
P. decaturense			605	10		499				10		1104	4		628	6		476	10		1104
P. digitatum		3	154	4	11	425		6	1	4	8	578	4	2	8		12	571	4	14	579
P. echinulatum				4			2			2						4			4		
P. expansum	10		29690	14		3848			25599	24		7939	4		24067	20		9471	24		33538
P. exsudans				2		4196	2		3929			267			4196	2			2		4196
P. frequentans	2		1073	2		681	4		1			1753	2		1708	2		46	4		1754
P. georgiense		10						9			1			7			3			10	
P. glabrum	8		560	6		13320	10		10045	4		3835	6		13686	8		194	14		13880
P. griseofulvum	16		828	2		3652				18		4480	8		2537	10		1943	18		4480
P. griseopurpureum			954			921						1875			921			954			1875
P. guanacastense				4			4									4			4		
P. hetheringtonii	2		6946	4		8393			3814	6		11525	4		4350	2		10989	6		15339
P. infrapurpureum	2		8	2			4					8	2			2		8	4		8
P. janczewskii				2		4				2		4				2		4	2		4
P. janthinellum				2			2									2			2		
P. javanicum	2		811	2		77	2			2		888			170	4		718	4		888
P. jejuense	2						2						2						2		
P. koreense	20		11373	14		62981	20		43778	14		30576	2		41294	32		33060	34		74354
P. limosum	2		904			713			1	2		1616			564	2		1053	2		1617
P. magnielliptisporum	2		3567			292			2	2		3857	2		11			3848	2		3859
P. mallochii	2						2						2						2		
P. maximae		3	1012	6	3	38	2	4		4	2	1050	2	3	2	4	3	1048	6	6	1050
P. menonorum	10		25729			35903	4		6559	6		55073			45051	10		16581	10		61632

P. mexicanum			20090	2		799			20089	2		800			14714	2		6175	2		20889
P. multicolor		2		2	3	793	2				5	793		2	648	2	3	145	2	5	793
P. nalgiovense	4		283				4		283							4		283	4		283
P. ochrochloron						45						45			45						45
P. olsonii			6910			1			6906			5			2420			4491			6911
P. ornatum			31									31			31						31
P. ovatum			147						147									147			147
P. oxalicum	54	65	13429	48	32	4688	34	69	670	68	28	17447	16	53	12738	86	44	5379	102	97	18117
P. paneum			730	10		1128	6			4		1858	2		1250	8		608	10		1858
P. paxilli			825						824			1						825			825
P. piscarium	4			2		16	2			4		16	2			4		16	6		16
P. polonicum			12896	18		4579			11509	18		5966	2		13164	16		4311	18		17475
P. radicicola			8			1000						1008			1008						1008
P. raperi	92	7	17996		237	29299	84	239	8759	8	5	38536	12	110	20204	80	134	27091	92	244	47295
P. rubefaciens						54454			54116			338			22581			31873			54454
P. rubens	2			8		86	6			4		86	8		4	2		82	10		86
P. samsonianum			296	2						2		296	2		296				2		296
P. sanguifluum			1470						1470						1			1469			1470
P. solitum			1680	4		871				4		2551			1025	4		1526	4		2551
P. steckii			7251	8		16334	2		230	6		23355	2		6457	6		17128	8		23585
P. sumatrense	8	3	560	4	1	133	10	3		2	1	693	10	1	262	2	3	431	12	4	693
P. terrigenum	4	5	16584	4	5	45939	4	2	6	4	8	62517	6	5	34188	2	5	28335	8	10	62523
P. ulaiense						343						343			343						343
P. velutinum	2		7173			1476	2		1			8648	2		2734			5915	2		8649
P. viticola	2	23	2274		2		2	22	2274		3		2	21			4	2274	2	25	2274
P. westlingii			3	4		393	2		1	2		395	2		395	2		1	4		396

P. yarmokense			820			1			820			1						821			821
Penicillium sp. 01	18		3509	10		15976	14		8047	14		11438	16		10737	12		8748	28		19485
Penicillium sp. 08	4	2	404	4	12	2105	4	7		4	7	2509	4	3	1270	4	11	1239	8	14	2509
Penicillium sp. 10				2			2						2						2		
Penicillium sp. 11	2		83							2		83			83	2			2		83
Penicillium sp. 13	2		4558	2		4587				4		9145			3793	4		5352	4		9145
Penicillium sp. 14	2		246			75	2					321			7	2		314	2		321
Penicillium sp. 16			8	4		439				4		447	4		447				4		447
Penicillium sp. 17	2						2						2						2		
Penicillium sp. 21						381						381			381						381
Penicillium sp. 22			168			790			958									958			958
Penicillium sp. 23			299						299						299						299
Penicillium sp. 24			43						43									43			43
Penicillium sp. 25			630			1			630			1			1			630			631
Penicillium sp. 26			45						45						28			17			45
Penicillium sp. 27			28						28									28			28
Penicillium sp. 28		12			4			10			6			8			8			16	
Penicillium sp. 29		13			9			16			6			6			16			22	
Penicillium sp. 33		18			40			33			25			36			22			58	
Penicillium sp. 34		19			12			14			17			20			11			31	
Penicillium sp. 36		8			94			79			23			94			8			102	
Penicillium sp. 38		3			10			8			5			4			9			13	
Penicillium sp. 39		12			9			7			14			8			13			21	

Supplementary Table 3 Growth rate of *Penicillium* species at different temperature on malt extract agar

Species	Strain	Growth rate (mm)				
		10°C	15°C	20°C	25°C	30°C
P. allii	SFC20151118-M09	10.3	23.0	32.0	39.0	16.3
P. allii-sativi	SFC20151118-M13	8.7	20.7	28.3	33.7	31.7
P. antarcticum	SFC20150915-M01	10.0	23.7	32.7	46.3	7.0
P. atramentosum	SFC20150402-M34	8.3	14.3	14.3	17.7	8.7
P. aurantioviolaceum	SFC20150303-M06	9.3	22.0	32.3	42.0	38.0
P. bialowiezense	SFC20151014-M03	11.0	17.3	22.7	24.3	0.0
P. bissettii	SFC100822	6.3	19.0	28.0	35.3	35.7
P. brasilianum	SFC20150303-M10	5.7	12.7	16.3	30.0	42.7
P. brevicompactum	SFC20151014-M11	7.0	16.0	22.0	22.0	3.0
P. caperatum	SFC20151118-M06	3.0	12.0	16.7	23.7	35.3
P. charlesii	SFC20150915-M13	3.0	8.0	15.3	18.0	9.0
P. chrysogenum	SFC20151014-M02	9.3	22.0	26.3	34.7	38.3
P. citrinum	SFC20151118-M02	3.0	9.0	17.0	26.0	30.3
P. commune	SFC20151118-M04	13.0	26.7	33.0	30.7	13.3
P. coprophilum	SFC20150303-M11	6.7	13.3	24.0	27.3	29.3
P. copticola	SFC20150303-M12	3.0	10.0	21.3	28.3	20.7
P. cremeogriseum	SFC20151014-M15	3.0	11.7	23.3	33.7	45.3
P. crustosum	SFC20151118-M08	12.0	24.0	31.7	34.3	28.0
P. daejeonium	SFC20151014-M05	5.3	14.0	22.0	28.3	12.7
P. decaturense	SFC20150303-M14	6.0	17.3	27.0	33.0	9.7
P. digitatum	SFC20151014-M04	4.7	16.0	24.3	25.0	6.0
P. echinulatum	SFC20151014-M09	11.3	23.7	30.3	35.0	13.0
P. expansum	SFC20150402-M38	11.0	19.7	27.7	34.7	3.3

P. exsudans	SFC100716	4.7	13.3	20.3	27.7	29.0
P. frequentans	SFC20150915-M09	7.7	19.7	27.0	35.0	24.7
P. glabrum	SFC20150915-M02	9.7	19.3	28.3	34.3	34.0
P. griseofulvum	SFC20151118-M01	7.7	17.3	24.0	26.3	24.7
P. guanacastense	SFC100711	5.3	10.0	16.7	25.0	31.3
P. hetheringtonii	SFC20151014-M08	3.3	12.0	17.7	31.0	32.0
P. infrapurpureum	SFC20150915-M12	4.0	8.3	13.3	16.0	7.7
P. janczewskii	SFC20151014-M10	4.3	11.3	18.0	26.0	19.3
P. janthinellum	SFC20151118-M12	3.3	12.7	20.7	29.3	35.0
P. javanicum	SFC20151118-M07	8.3	16.0	22.0	21.3	0.0
P. jejuense	SFC20150402-M41	7.3	17.7	29.7	39.7	14.0
P. koreense	SFC20151014-M13	3.0	9.0	20.3	28.7	33.3
P. limosum	SFC20151118-M05	3.0	13.0	26.3	38.0	47.0
P. magnielliptisporum	SFC20151118-M03	10.0	7.7	10.7	11.3	6.3
P. mallochii	SFC20150915-M03	4.0	11.3	20.0	29.0	23.0
P. maximae	SFC20151014-M14	3.0	12.0	21.0	30.0	32.7
P. menonorum	SFC20150915-M07	0.0	3.0	9.0	13.0	19.0
P. mexicanum	SFC20151014-M01	8.7	16.3	15.7	17.7	3.0
P. multicolor	SFC20151118-M10	3.0	7.7	12.3	17.0	13.0
P. nalgiovense	SFC20150402-M42	5.7	17.0	28.0	34.3	17.0
P. oxalicum	SFC20150915-M10	5.0	20.0	32.3	43.3	50.0
P. paneum	SFC20150915-M14	10.0	25.3	38.3	44.7	47.7
P. piscarium	SFC20151014-M16	5.0	16.0	26.3	37.3	44.7
P. polonicum	SFC20151014-M06	10.0	22.0	32.0	33.7	21.3
P. raperi	SFC20150915-M08	8.7	20.7	28.0	35.3	36.0
P. rubens	SFC20151014-M07	9.7	19.0	25.3	39.0	37.0
P. samsonianum	SFC100899	8.0	16.0	23.0	23.3	9.3

P. solitum	SFC100989	11.7	20.0	25.0	30.0	3.0
P. steckii	SFC20150303-M17	3.0	9.0	19.7	28.0	23.7
P. sumatrense	SFC20150915-M04	3.7	14.3	24.7	31.3	17.3
P. terrigenum	SFC20150915-M06	3.0	12.3	20.7	32.0	27.0
P. velutinum	SFC20150915-M11	6.0	19.0	31.3	45.0	50.0
P. viticola	SFC20150915-M05	6.7	14.7	24.0	29.3	9.0
P. westlingii	SFC20151118-M11	7.7	16.0	19.7	20.3	9.3
Penicillium sp. 01	SFC20150303-M09	5.3	13.0	25.0	33.0	31.7
Penicillium sp. 08	SFC100917	6.0	17.0	24.3	35.3	42.7
Penicillium sp. 10	SFC100725	0.0	8.3	18.7	19.3	11.0
Penicillium sp. 11	SFC101038	0.0	3.0	9.0	13.0	19.0
Penicillium sp. 13	SFC100985	4.7	13.7	20.3	32.7	40.7
Penicillium sp. 14	SFC100817	7.7	15.7	27.0	33.7	19.7
Penicillium sp. 16	SFC100919	3.0	13.7	21.7	36.7	43.0
Penicillium sp. 17	SFC100747	3.0	10.3	22.3	28.3	37.7

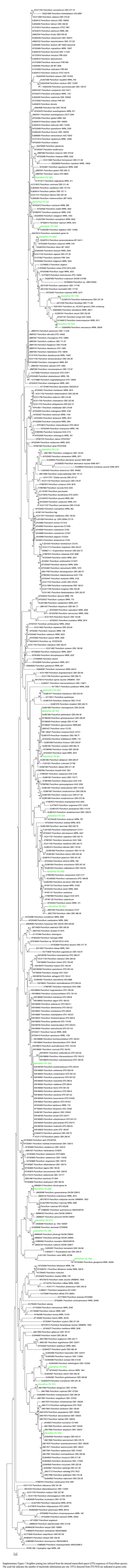

Supplementary Figure 1 Neighbor joining tree inferred from the internal transcribed spacer (ITS) sequences of *Penicillium* species. The scale bar indicates the number of nucleotide substitutions per site. OTUs detected from ITS-NGS are indicated in green color.

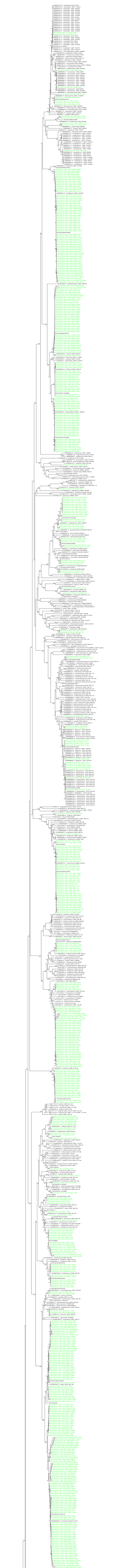

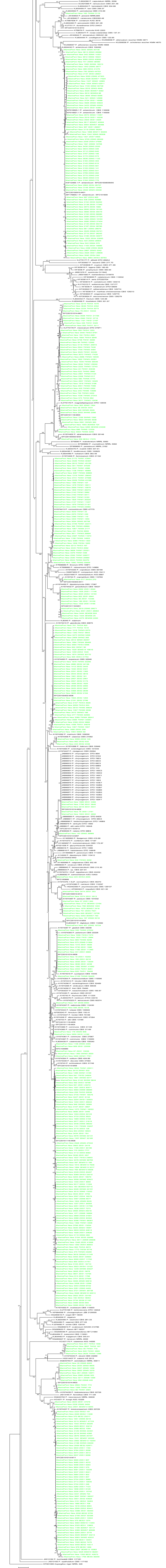

Supplementary Figure 2 Neighbor joining tree inferred from partial β-tubulin (BenA) sequences of *Penicillium* species.
The scale bar indicates the number of nucleotide substitutions per site. OTUs detected from BenA-NGS are indicated in green color.
